# Prevalence of Second Root and Root Canal in Mandibular and Maxillary Premolars Based on Two Classification Systems in Sub-Population of Northern Region (Saudi Arabia) Assessed Using Cone Beam Computed Tomography (CBCT): A Retrospective Study

**DOI:** 10.3390/diagnostics13030498

**Published:** 2023-01-29

**Authors:** Sultan Meteb Alshammari, Kiran Kumar Ganji, Amjad Abdulrahman Albulayhid, Akram Mojidea Alshammari, Khalid Hamoud Raja Alhassan, Munahi Abdullah Rushdallah Alsirhani

**Affiliations:** 1 Department of Preventive Dentistry, College of Dentistry, Jouf University, Sakaka 27343, Saudi Arabia; 2 Department of Periodontics & Implantology, Sharad Pawar Dental College, Datta Meghe Institute of Higher Education & Research, Sawangi (Meghe), Wardha 442107, India

**Keywords:** root canals, roots, cone beam computed tomography, maxillary premolars, mandibular premolars

## Abstract

The objective of this paper is to assess the prevalence of a second canal in maxillary and mandibular premolars based on two classification systems of root canal morphology using Cone beam computed tomography (CBCT) images. A total of 286 CBCT scans from the archive of the Radiology department of a hospital were assessed for the presence of a second canal in maxillary and mandibular premolars. The canal configuration and its assessment was undertaken using Vertucci and Ahmed’s classification. A Chi-square test was used to test the significance of the difference between gender and age. A total of 286 premolars were examined (217 mandibular premolars and 69 maxillary premolars); of these, 173 teeth (60.5%) were from males and 113 were from females (39.5%). Some 62% of maxillary left first premolars had two roots, followed by maxillary right first premolars (47%), and then maxillary left second premolars (30%) and maxillary right ones (27%), respectively. Type IV Vertucci were seen in maxillary premolars, while type I were ordinarily seen in the included mandibular premolars. Surprisingly, Vertucci type III was only found in mandibular left first premolars at a frequency of 2%. One orifice with two separate canals and two orifices of two distinct canals with two portals of exit were predominantly noticed with maxillary first premolars (^2^ FP B^1^ P^1^) in 73% and 81%, respectively, followed by (^1^ FP ^2^) 19%. The prevalence of a second canal in maxillary and mandibular premolars was low in the investigated premolars in comparison to the premolars that had just one root and canal, as assessed based on Vertucci and Ahmed’s root canal system classification.

## 1. Introduction

Successful endodontic treatment is the primary aim of any clinician who treats pulpally involved teeth. The efforts of clinicians can be more effective if they have a rigorous knowledge of internal tooth anatomy (for example, a second canal, canal bifurcation, etc.), which subsequently leads to easy root canal treatment assisted by the appropriate orifice detection. Furthermore, this results in proper cleaning and shaping alongside the good obturation of root canals, avoiding any kind of mishaps. The complexities of such structures necessitate the further understanding of root canal anatomy and factors that play an important role in the variability of root canal anatomy [1].

Regular radiographs were used to provide an evaluation of internal tooth anatomy but were of limited value. Indeed, such 2D images are subjected to various superimpositions [2]. On the other hand, a sophisticated technology such as cone beam computed tomography (CBCT) is well established as being highly sensitive. In fact, it is used to indicate the proper way of studying root canal anatomy via 3D images, overcoming all means of structure superimposition, which can guide clinicians towards a suitable diagnosis and, subsequently, the appropriate intervention. E-Vol DX CBCT software has recently been developed for use in endodontics. It starts from diagnosis until achieving the desired treatment plan, whereby clinicians are able to detect any defects and provides the ability to study root canal complexities more efficiently [3]. CBCT also aids in the detection of caries, especially cavitated/non-restored teeth, which should be nonmetallic, as metallic restorations produce an artifact. It similarly aids in assessing periapical lesions, differentiating between fluid-filled and sold lesions, determining the prevalence of a second mesiobuccal (MB2) canal and other accessory canals or further dental anomalies such as dens invaginatus and the presence and extent of root fractures [4,5,6].

Weine et al. were the first to classify root canal configurations inside a single root into three categories based on the pattern of root canal division along its path from the pulp chamber to the root apex [7]. Sometime after this, Vertucci et al. proposed a new classification based on investigations done on the maxillary second premolar, and identified a total of eight configurations [8]. Kartal N et al. then investigated mandibular anterior teeth, and found two new root canal configurations. Furthermore, Kartal N et al. published another study that found type II Vertucci to be divided into two subgroups: Type IIa and Type IIb [9]. Based on the number of roots and the number of canals, Zhang, R. et al. investigated mandibular molars only, and provided seven variations of root canal anatomy [10]. Silva EJNL et al. then found three more variations, bringing the total to ten variations [11]. Recently, Kottoor et al. and Albuquerque et al. proposed a new nomenclature for root canal anatomy classification for maxillary and mandibular molars [12]. Several studies conducted among the Turkish, Chinese, Iranian, Jordanian, Malaysian and American populations revealed differences in the root canal morphology of permanent anterior and posterior teeth [13,14,15,16,17]. It is important to mention that for many years the most commonly used root canal classification system is the Vertucci and supplemental configuration types [18,19,20]. Indeed, those were found to be non-comprehensive, as Ahmed HMA et al. reported various shortcomings of Vertucci’s classification system. For instance, shortcomings included the defining of the number of roots, the outline of the pulp chamber, and the lack of clarity with regard to multi-rooted teeth, predicting the complexity of root canal anatomy. These factors are of crucial importance and need to be fully understood by a clinician before undertaking a root canal treatment in order to provide a successful intervention [21].

Given the above, Ahmed HMA et al. proposed a new classification system that can be adapted to categorize root and root canal configurations in an accurate, simple and reliable manner. It can be used in research and clinical practice, as well as in training [22].

There is a dearth of studies in the literature from Saudi Arabia with regard to the study of the root canal morphology of maxillary and mandibular premolars using CBCT images based on both Vertucci and Ahmed’s classification system. Therefore, the aim of this study is to assess the prevalence of the second canal in maxillary and mandibular premolars based on two classification systems of root canal morphology using CBCT images.

## 2. Methods

This study was carried out after obtaining approval by the Local Committee of Bioethics of Jouf University (Approval no. 8-04-43). CBCT images were obtained from the archive of the Department of Radiology section of the College of Dentistry, Jouf University. CBCT scans had already been made for patients for diagnostic purposes. Images taken by SCANORA 3Dx (Nahkelantie 160, Tuusula, Finland) with standard operating specifications (90 KV and 10 mA), were used for the analysis of second canal and root canal morphology in both jaws with a medium field of view (FOV = 80 × 100), where a standard resolution mode (voxel size of 0.25 mm) was designated. The allocated complete scan time comprised a 360° rotation of X-ray receptor assembly around the static patient.

**Figure 1:**
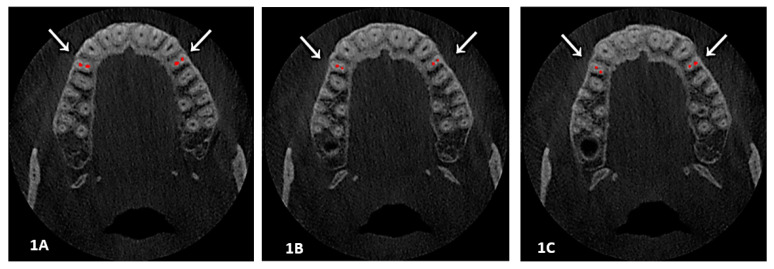
(**1A**, **1B** and **1C**): An axial view showed a root canal system at different levels; here is an example of two canals with two foramen at three levels, Section (**1A**) at orifice, while Section (**1B**) is at the coronal-middle third, and Section (**1C**) is at the foramen level. Classification codes came as Vertucci IV, ^2^ FP B^1^ P^1^**.**

**Figure 2:**
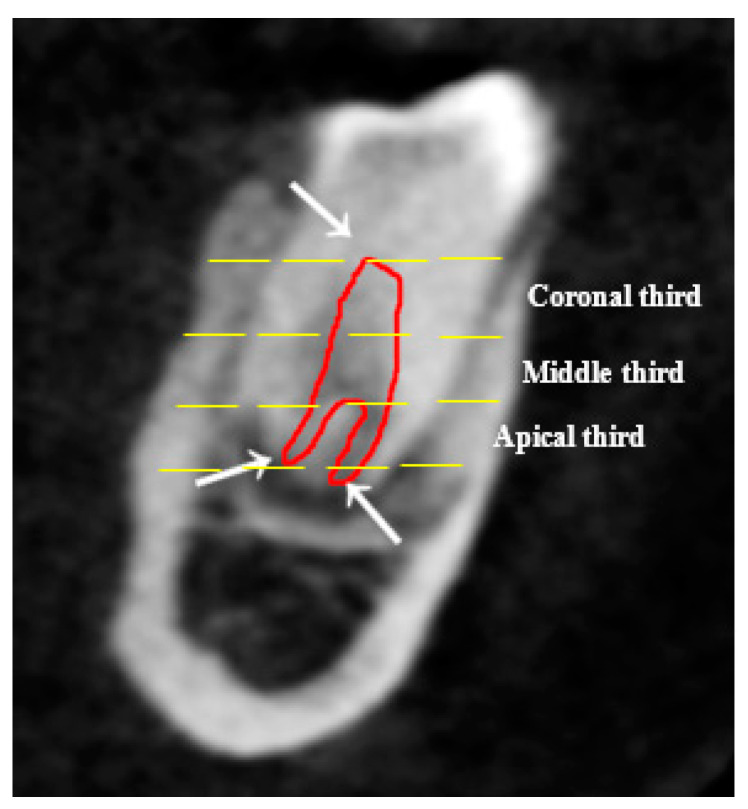
The sagittal view showed the root canal from orifice to portal of exit, one canal at orifice/coronal and the middle thirds, which split into two foramina apically; it is an example of Vertucci V and ^1^ FP ^1,1,2^ based on both Vertucci and Ahmed’s classification system of root canals.

Two sections (axial and sagittal views) of each CBCT image with a significant number of slices were analyzed. The root canal tracing using an Axial view helped in studying root canal morphology in respect to Ahmed’s classification from the orifice to the foramen, course and number of canals at three levels: orifice, coronal third of root and portal of exit. Furthermore, the sagittal view was used for detecting a root canal configuration and applying Vertucci’s patterns (see Figure 1A–C and Figure 2).

### 2.1. Inclusion Criteria

We included patients above the age of 14 from both genders who had maxillary and mandibular premolars with roots fully formed and no endodontic intervention or prosthesis.

### 2.2. Exclusion Criteria

We excluded edentulous arches, patients with missed upper and lower premolars, or who had already been endodontically treated and teeth where apices were not fully formed or had blurred CBCT images, as well as syndromic or systematically diseased patients affecting premolar formation.

### 2.3. Statistical Analysis

The measured values for canal configurations were analyzed using Statistical Package for Social Sciences software version 25 (IBM, Chicago, IL, USA). Descriptive statistics were performed to assess the distribution of age and gender. A chi-square test of independence was used to assess the significance between the number of roots, number of canals, Vertucci’s pattern, and Ahmed’s classification pattern. *p* < 0.05 was considered to be statistically significant. The test-retest reliability index was calculated to assess the consistency of outcome variables, where in Vertucci’s pattern was considered as test and where in Ahmed’s classification pattern was considered as retest.

## 3. Results

**Figure 3:**
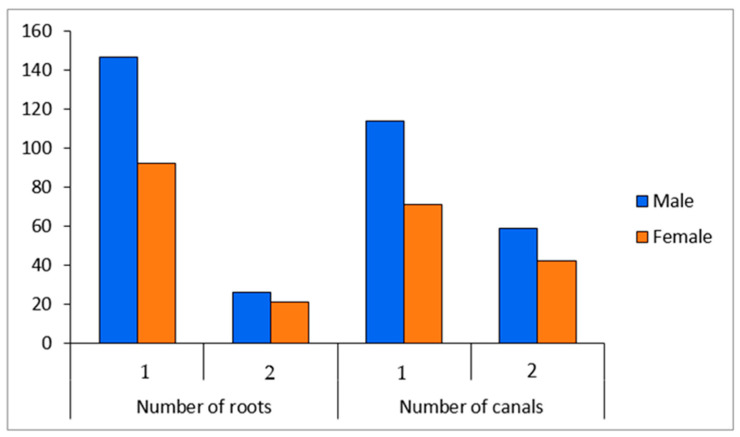
Number of roots and canals in relation to gender.

**Figure 4:**
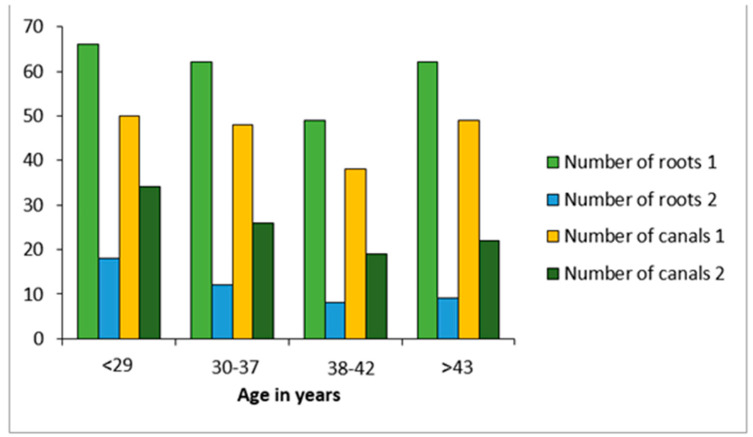
Number of roots and canals in relation to age.

A total of 286 premolars were examined (217 mandibular premolars and 69 maxillary premolars); of these, 173 teeth (60.5%) were from males and 113 were from females (39.5%). Based on Vertucci and Ahmed’s classification patterns, the examined teeth had a variety of root and root canal numbers and configurations. Two roots and two root canal systems were found among young people aged 15 to 35 years, with a slight male predilection (see Figure 3 and Figure 4).

One root was found to be predominant in the maxillary right and left second premolars (73% and 70%, respectively), whereas 53% were maxillary right first premolars, followed by 38% of maxillary left first premolars. Among these, 62% of maxillary left premolars had two roots, correspondingly followed by maxillary right premolars (47%), maxillary left second premolars (30%) and maxillary right premolars (27%). The majority of mandibular premolars had a single root, with mandibular right first and mandibular left second premolars comprising 93%. This was followed by mandibular right second premolars at 92% and mandibular left second premolars at 89%. Two root mandibular premolars presented at 11% with mandibular left second premolars, followed by mandibular right second premolars (8%). and 7% among both mandibular right first premolars and left second premolars. In terms of root canals, maxillary left first premolars had the greatest percentage of two canals at 82%, followed by maxillary left second premolars at 76%, whereas 74% were maxillary right first premolars and 58% were maxillary right second premolars. A single canal was found in the majority of mandibular premolars (71–82%), while two canals were found in 18–29% of included mandibular premolars (see Table 1).

The highest percentage of a single root with a single canal seen with mandibular right second premolars was 93% and 82%, respectively. On the other hand, the highest percentage of two roots and two canals seen in maxillary left first premolars was 62% and 82%, respectively. Concerning root canal configuration, Vertucci type IV was remarkably seen in maxillary premolars, while type I was ordinarily seen in the included mandibular premolars. Surprisingly, Vertucci type III was only found in mandibular left first premolars at 2% (see Table 1).

According to Ahmed’s formula for double-rooted teeth (^2^TN R1 ^O, C, F^ R2 ^O, C, F^), one root (92%) with a canal from the orifice to foramen (^1^ 35 ^1^) was remarkably seen with mandibular left second premolars (82% and 86%). One orifice with two separate canals and two orifices of two distinct canals with two portals of exit were predominantly seen with maxillary first premolars (^2^ FP B^1^ P^1^ ) at 73% and 81%, followed by ^1^ FP ^2^ at 19%. Furthermore, ^1^ SP B^1^ P^1^ and ^1^SP ^1-2^ codes were commonly seen in maxillary second premolars (see Table 1). 

Test-retest reliability measured for both classification systems presented the stability of outcome variables across time (*p* < 0.05, r = 0.86). Overall, the reliability coefficient was 0.81, thus it refers to the fraction of the total variance, which is not attributable to measure errors.

## 4. Discussion

This study reports on the prevalence of second root and root canals in mandibular and maxillary premolars and the configuration of a root canal system based on Vertucci and Ahmed’s classifications in a sub-population of the northern region of Saudi Arabia using CBCT. The need for an understanding of root anatomy and canal numbers is evidently critical for successful endodontic therapy [23]. Recent studies have found significant differences in the morphology of the root and root canal of mandibular first and second premolars among the Saudi subpopulation. Furthermore, endodontic treatment is challenging, particularly with mandibular first premolars, due to the presence of many variations and the restricted accessibility to the second canal [24,25]. Age and gender [26,27], as well as research design, canal identification methods, and ethnic differences all contribute to such differences [28].

Root canal variations are fairly prevalent according to several researchers [23,28,29]. In this study, we found that most young people aged 15 to 35 years with a slight male predilection have two roots and two root canal systems (see Figure 3 and Figure 4). Our findings revealed that the majority of maxillary second premolars had just one root and canal, which is consistent with prior research that established that 82.1% of maxillary second premolars had only one root and canal [23,25]. In a recent study, Al-Zubaidi S.M. et al. [30] found that 60.4% of maxillary second premolars had a single canal; surprisingly, our results revealed that 58% to 76% of maxillary right second premolars had two canals.

In the literature, two roots were identified in 70% of maxillary first premolars among the Polish population [28], which is in agreement with comparable percentages of 70.1% in the Kosovan [31], 73.3% in the Ugandan [32], 71.7% in the Saudi [33] and 61.3% in the Turkish [34] populations. Our results showed that maxillary right and left first premolars had two roots at 47% and 62%, respectively. Comparatively, our findings were to a lower extent than other studies previously cited. An Indian population, on the other hand, was found to have a greater percentage of double-rooted maxillary premolars (91.7%) [35]. Three roots of the maxillary first premolar are uncommon, which corresponds to what we discovered at 0% [28].

With respect to mandibular premolars, our data clearly showed that the majority of patients had one root with a single canal, ranging from 89% to 93%, while two rooted mandibular premolars ranged from 7% to 11%. Similarly, a study published in 2019 on the Saudi population [24,36] found that mandibular premolars exhibited one root in 96.4% of first premolars and 95.6% of second premolars. Similarly, many studies have reported one rooted mandibular premolars as comprising 98% of the Thai population [37], 98–100% of the western Chinese population, 100% of the Spanish population [38], and 85.7–94.8% of the Iranian population [39]; 97% was reported by Cleghorn BM et al. [40,41]. In terms of root canal numbers, maxillary premolars showed a larger percentage than mandibular premolars, regardless of whether they were first or second, or right or left. In this study, we found that 18–42% of maxillary premolars had a single canal, whereas 58–82% had two canals. 

Maxillary first premolars with two canals had a greater proportion (74–82%) than maxillary second premolars (58–76%). When comparing the first and second maxillary premolars on the right and left sides, we noticed that the left side had a larger percentage of two canals (76–82%) than the right side (58–74%). Meanwhile, single canals were found in 71–82% of mandibular premolars and two canals were found in 18–29% of mandibular premolars. We would emphasize that the distribution of the number of canals is almost the opposite of maxillary premolars where two canals predominated. When compared to prior cited studies, single canals were found in 87.1–97.2% of mandibular premolars in western China [42], in 83.3% of the Spanish population [38], and in 63.9–78.3% of the Iranian one [39]. Blacks (32.8%) had the highest prevalence of mandibular premolars with two or more canals, whereas Caucasians had the lowest prevalence at 13.7% [43].

In this study, a Vertucci type IV configuration of the root canal system was shown to be prevalent in 81% of maxillary left first premolars, which is consistent with Shadia Maghfuri et al.’s findings [44]. This is nearly similar to prior studies, which found 74.7% in a Kuwaiti population [45], 79.7% in a Jordanian population [29], and 77% in a Turkish population [34]. However, this varied in other studies, all of which used CBCT analyses ranging from 51% to 76% [26,46,47].

In terms of root canal configuration, our results revealed that Vertucci type I was prevalent among mandibular premolars; it showed 79% and 80% between second premolars followed by 71% and 76% in first premolars. To some extent, a recently published study conducted for a Thai population showed quite similar results, at 98% and 63.1% [37]. Although the majority of mandibular premolars were Vertucci type I, we found that mandibular first premolars were also classified as Vertucci type IV (14%–17%), which is inconsistent with a number of studies that showed Vertucci type V among mandibular first premolars at 21.91–24% and 28.5% [37,48,49,50,51,52,53]. Surprisingly, our studied population showed only the first five types of Vertucci’s pattern, while in the literature many studies reported the presence of all patterns except for type VI, which was seldom or not found [37,48,49,50,51,52,53]. Many studies published earlier found no single example of Vertucci type III; our study proved its presence in a few cases by finding only in the mandibular left first premolar by 2% [8,29,54,55,56,57]. 

As many studies have established the accuracy of Ahmed’s classification of the root canal system and maxillary and mandibular premolars in order to assess the most prevalent classification code of the root canal system, type 1 SP 1 was most frequently seen with mandibular left second premolars (82% and 86%). Moreover, type 2FP B1P1 was detected at 73% and 81% in maxillary first premolars, followed by type 1 FP 2 in 19% of maxillary left first premolars. As a result, there were two rooted premolars in a Saudi population with two distinct canals from orifices to foramen, which were most commonly seen in maxillary premolars. Similarly, two studies conducted among Egyptian [19] and Polish populations [28] yielded the same results. Our findings demonstrate that type 1SP B1 P1 and 1SP 1-2 codes were ordinarily seen in maxillary second premolars and were in agreement with a study published in 2019 by Saber et al. [58] In contrast, a study conducted on a South African subpopulation by Buchanan GD et al. [59] established that 1MP1 was the most common code in maxillary second premolars. With respect to the prevalence of the second canal, many studies by Atieh MA [28], Elkady AM et al [28], Maghfuri S et al. [44], Alqedairi A et al. [60], and Al-Zubaidi SM et al. [55] found that the majority of maxillary first premolars had two roots among the Saudi population, which is similar to our findings. Regarding mandibular premolars, the majority of mandibular premolars in the Saudi population had one root with a single canal. Our results are in agreement with Alfawaz H et al. [28] and Al-Zubaidi SM et al.’s [61] studies. Our limitation was that the result cannot be generalized as it was performed on a specific geographic area. We recommend conducting multicenter studies. We recommend conducting multicenter studies where the results can be generalized.

## 5. Conclusions

The prevalence of the second canal in maxillary and mandibular premolars was low among investigated premolars in comparison to premolars that had just one root and canal as assessed based on Vertucci and Ahmed’s root canal system classifications. Knowledge of the second canal in maxillary and mandibular premolars enhances the development of comprehensive endodontic care.

## Figures and Tables

**Table 1:** Comparison of roots and canal configuration in according to Vertucci and Ahmed’s classifications.

Tooth No.	Number of Roots		Number of Canals		Vertucci Pattern					Ahmed’s Classification Pattern						*p*-Value
										Orifice		Canal		Foramen		
	1	2	1	2	Type I	Type II	Type III	Type IV	Type V	1	2	Common	Buccal & Palatal	Common	Buccal & Palatal	**<0.05**
**#14**	53%	47%	26%	74%	32%			68%		27%	73%	27%	73%	27%	73%	
**#15**	73%	27%	42%	58%	47%	47%		6%		43%	57%	43%	57%	43%	57%	
**#24**	38%	62%	18%	82%	19%			81%		19%	81%	19%	81%	19%	81%	
**#25**	70%	30%	24%	76%	30%	6%		64%		24%	76%	24%	76%	24%	76%	
**#34**	93%	7%	76%	24%	76%	2%	2%	14%	6%	83%	17%	81%	19%	81%	19%	
**#35**	92%	8%	80%	20%	80%	4%		13%	3%	86%	14%	82%	18%	82%	18%	
**#44**	89%	11%	71%	29%	71%	2%		17%	10%	73%	27%	74%	26%	74%	26%	
**#45**	93%	7%	82%	18%	79%	4%		13%	4%	85%	15%	82%	18%	82%	18%	

#, Specify tooth number.

## Data Availability

The data that supports the findings of the study will be made available upon reasonable request to the corresponding author subject to the author’s discretion.

## List of Abbreviations

Cone beam computed tomography (CBCT), two dimensions, MB2 second mesiobuccal canal, Vertucci Type IV-V, 2 FP B1 P1 ( two rooted (first premolar) one buccal canal one palatal canal, 1 FP 2(one root (first premolar) two distinct canals.
